# Interactions of Grazing History, Cattle Removal and Time since Rain Drive Divergent Short-Term Responses by Desert Biota

**DOI:** 10.1371/journal.pone.0068466

**Published:** 2013-07-16

**Authors:** Anke S. K. Frank, Chris R. Dickman, Glenda M. Wardle, Aaron C. Greenville

**Affiliations:** 1 Research Associate, Desert Ecology Research Group, School of Biological Sciences, The University of Sydney, Sydney, New South Wales, Australia; 2 Professor, Desert Ecology Research Group, School of Biological Sciences, The University of Sydney, Sydney, New South Wales, Australia; 3 Associate Professor, Desert Ecology Research Group, School of Biological Sciences, The University of Sydney, Sydney, New South Wales, Australia; 4 Research Assistant, Desert Ecology Research Group, School of Biological Sciences, The University of Sydney, Sydney, New South Wales, Australia; Bangor University, United Kingdom

## Abstract

Arid grasslands are used worldwide for grazing by domestic livestock, generating debate about how this pastoral enterprise may influence native desert biota. One approach to resolving this question is to experimentally reduce livestock numbers and measure the effects. However, a key challenge in doing this is that historical grazing impacts are likely to be cumulative and may therefore confound comparisons of the short-term responses of desert biota to changes in stocking levels. Arid areas are also subject to infrequent flooding rainfalls that drive productivity and dramatically alter abundances of flora and fauna. We took advantage of an opportunity to study the recent effects of a property-scale cattle removal on two properties with similarly varied grazing histories in central Australia. Following the removal of cattle in 2006 and before and after a significant rainfall event at the beginning of 2007, we sampled vegetation and small vertebrates on eight occasions until October 2008. Our results revealed significant interactions of time of survey with both grazing history and grazing removal for vascular plants, small mammals and reptiles. The mammals exhibited a three-way interaction of time, grazing history and grazing removal, thus highlighting the importance of careful sampling designs and timing for future monitoring. The strongest response to the cessation of grazing after two years was depressed reproductive output of plants in areas where cattle continued to graze. Our results confirm that neither vegetation nor small vertebrates necessarily respond immediately to the removal of livestock, but that rainfall events and cumulative grazing history are key determinants of floral and faunal performance in grassland landscapes with low and variable rainfall. We suggest that improved assessments could be made of the health of arid grazing environments if long-term monitoring were implemented to track the complex interactions that influence how native biota respond to grazing.

## Introduction

On a global scale, the managed grazing of livestock occupies more land than any other human enterprise and covers over a quarter of Earth’s land surface. Livestock grazing is particularly prevalent in arid and semi-arid rangelands that are dominated by grasses and shrubs [1], but is expanding increasingly into other habitats such as forest and woodland as these are converted into pasture [2]. The addition of domestic stock to arid grasslands is likely to alter the abundance, diversity or composition of the existing biota, generating debate about the magnitude and kinds of responses that might be expected by these biota [3]–[6]. Resolving this debate is difficult. On the one hand, biotic change could be measured after introducing livestock to new areas of arid grassland, but few sites now exist where livestock grazing has not previously occurred. On the other hand, currently-grazed areas could be destocked and the responses of biota then compared in removal and control sites. A challenge with the latter approach, however, is to account for other factors that might also drive biotic change [7]. Three key factors that may act singly or in combination are likely to be particularly influential in altering the composition or functional aspects of arid grassland systems.

Firstly, differences in grazing history, or the cumulative effects of grazing pressure over time, can affect arid grassland environments. For example, prolonged grazing pressure may compound biotic changes such as loss of soil crusts or reinforce feedback mechanisms via the loss or compaction of topsoil [7], [8]. Continued grazing pressure, even at low stocking rates, may therefore constitute a greater level of disturbance than one-off pulses of grazing, providing some support for the idea of rotational grazing [9].

Secondly, there may be threshold effects of grazing, non-linear or synergistic influences that result from changes at one trophic level cascading through to higher trophic levels. If, for example, there are continuous losses of palatable plant species that are directly consumed by stock, declines of these species may affect granivores via diminished seed resources when certain thresholds have been exceeded [8]. Non-reversible changes are another avenue for synergistic outcomes resulting from trophic interactions. Thus, if grazing pressure has occurred over a prolonged period and some of the original species have become locally extinct, the removal of grazers will not lead to a return to the original levels and composition of the biota [e.g. 10,11]. Therefore, we can expect interactions between historical levels of grazing and the removal of grazing pressure in how they influence the abundance and composition of the arid grassland biota.

Finally, any environmental and consequential biotic changes due to grazing must be understood in the context of the temporal dynamics of the boom and bust periods that characterise arid grasslands [12]. Most importantly, the species composition of arid grassland systems is dependent on time-since-flooding-rains, as major rainfall events drive the productivity of these environments [13]. During extended drought periods the abundance of most flora and fauna is low and many naturally rare species are absent except in scattered refugia where they persist. In these conditions, any biotic differences that do exist between grazed and ungrazed areas will be minimal and difficult to quantify. For these several reasons, livestock removal experiments that aim to uncover the effects of grazing on native biota need to account for historical grazing pressure in the study region and to be carried out over multiple years that include at least one major rainfall event.

Our research took advantage of a unique opportunity to study the interactions of recent cattle removal and historic grazing pressure on arid grassland flora and fauna just before and until 18 months after a major rainfall event in the Simpson Desert, central Australia. Here, two properties shared a similar history of cattle grazing that was more intense in the east than in the west owing to the difficulty of access to the western region that required over 100 sand dunes to be crossed. One of these properties was gazetted as a conservation reserve just before we commenced our study. This allowed us to set up an orthogonal repeated measures design that explored the interactive effects of recent cattle removal and historic grazing pressure on biota in this arid boom and bust system.

We address the following questions:

1. Are there effects of historical grazing pressure on desert biota?
2. Are there effects of recent cattle removal on desert biota?
3. How and when do these effects influence vegetation cover, flower and seed abundance and the abundance, species richness and diversity of desert fauna?
4. Do species within different functional groups respond differently to these effects?

Finally, we distil from our results some suggestions for the management of cattle and the protection of fauna (small mammals and reptiles) in arid grazing landscapes.

## Methods

### Study Area

The study was undertaken in the north-eastern Simpson Desert, central Australia. This extensive desert region covers about 170 000 km^2^ [14], and is characterized by parallel sand dunes that are up to 20 m high with swales on average 500–1000 m wide [15]. Median annual rainfall is 130–150 mm [15], but is extremely variable and locally patchy [16]. The annual mean temperature is 21°C–23°C [17] with summer temperatures often exceeding 46°C and winter temperatures falling to below freezing (–6°C) at night [15]. Vast hummock grasslands on poor sandy soils, dominated by hard spinifex (*Triodia basedowii*), occupy about 70% of the regional area, with smaller areas of tussock grassland, open woodland, shrubland and open areas with annual grasses and herbs also occurring [13], [18]. At least 12 mm of rain are required in a single rainfall event to trigger any growth of vegetation in spinifex grassland [19].

Cattle have been grazed intermittently in the eastern parts of the Simpson Desert since the mid-twentieth century, but more intensive and closely managed grazing has occurred only in the last 20–25 years. Over this period, improvements in vehicle technology have enabled easier access to the remote interior parts of the desert which are over 100 dune crests away from homesteads on the desert’s eastern fringe. Although cattle were nominally free to move westward with little obstruction from fences, it is likely that most stayed near the homesteads in the east due to the absence of water in the interior of the desert and because cattle avoid crossing dunes [20]. In addition to vehicular access, improvements in technology in the 1970s helped to maintain cattle in areas distant from the homesteads: access was gained to deep artesian water using bores run on diesel generators, and paddocks were established around the new watering points to confine the grazing herds for mustering operations [21].

**Figure 1:**
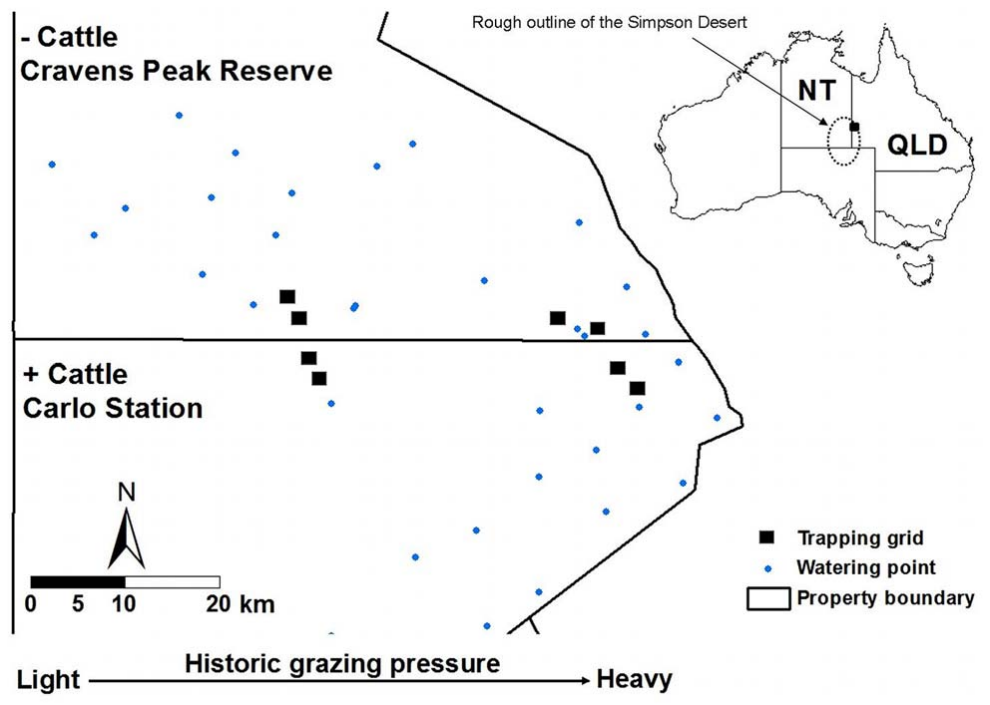
Location of trapping grids on Cravens Peak Reserve and Carlo Station. Trapping grids (not to scale), artificial watering points and the boundary fence between the two properties, are shown. Increasing historical grazing intensity from west to east is indicated by the arrow at the bottom. The inset shows the location of the Simpson Desert within Australia. NT = Northern Territory, QLD = Queensland.

Our sampling took place on two properties of similar size (>2000 km^2^) in this region, Carlo station (23° 29′S, 138° 32′E) and Cravens Peak Reserve immediately to the north (Fig. 1). Total numbers of cattle on both properties have fluctuated since the mid 1980s, with peaks after years of high rainfall and troughs due to destocking during droughts. However, overall densities have probably never exceeded the recommended stocking rate for the Simpson Desert Land System of one beast per 2.5 km^2^ [22] and, because of difficulty of access to the western parts of the properties, densities there were typically only 10–20% of those in the east (H. Jukes, Carlo station; L. Rule, Cravens Peak Reserve, pers. comms). The two properties shared a similar history of cattle grazing until 2005, when Cravens Peak was purchased by Bush Heritage Australia and all cattle were removed by the end of 2006. Other grazing mammals such as red kangaroos (*Macropus rufus*) and camels (*Camelus dromedarius*) occur, but at densities <0.05 animals/km^2^ [23], [24]; smaller grazers such as rabbits (*Oryctolagus cuniculus*) are present sporadically and in very low numbers [13], [25]. Wildfires generally occur every 25 years [18]. Prior to the beginning of this study, small areas (<10 ha) of both properties were subject to control burns to stimulate the growth of fresh grass and create breaks to prevent wildfires [26]. Investigation of the fire × grazing interaction was beyond our scope, and we confined our investigations to areas of long unburnt (>30 years) spinifex grassland which dominated the landscape throughout our study.

### Experimental Design

The difference in grazing history on the two properties (light grazing in the west compared to heavy grazing in the east) together with the difference in current land use (cattle absent from Cravens Peak Reserve but present on Carlo station) were used to define a balanced design with two crossed factors: grazing ‘treatment’ (2 levels: continuously grazed and cattle removed), and grazing ‘intensity’ (2 levels: ‘light’ and ‘heavy’ historical grazing pressure). To reduce the potential effects of latitudinal differences in climate, sample sites were set up close to either side of the boundary fence of the two properties, but far enough apart to be independent and not affected by the fence. In each of the four treatment areas, we established two plots to sample small vertebrates and vegetation (Fig. 1).

### Ethics Statement

The research was carried out under scientific license number WISP02994105 from the Queensland National Parks and Wildlife Service and with approval from the Animal Care and Ethics Committee of the University of Sydney (protocol numbers L04/4-2004/3/3895 and L04/1-2007/3/4510).

### Field Sampling

Sampling took place on eight field trips between May 2006 and October 2008, but due to logistical problems a full set of data was collected on only five of these occasions (May and September 2007 and April, June and October 2008). Before the study began conditions had been relatively dry with just 85 mm falling over the summer (November – February) of 2005–2006. Other than spinifex hummocks, vegetation in the study area was sparse and for some species the remaining above-ground tissue was dried and shrivelled, hindering identification beyond the genus level. Over the same period in 2006–2007, similarly heavy rains fell in the east and west of the study area (279 mm and 239 mm, respectively). There was a little follow-up rain in March 2007 in the historically heavily-grazed east (65 mm), but not enough to trigger further vegetation growth in the historically lightly-grazed west (11 mm) (on-site data obtained from automatic weather stations; Environdata, Warwick, Queensland). No further heavy falls were experienced, with the area drying from early 2007 until the end of the study. The summer of 2007–2008 brought less than 70 mm of rain in total, but slightly more again in the east (67 mm) than the west (32 mm).

To sample small vertebrates, a 1-ha trapping grid was established at each plot. Each grid contained 36 pitfall traps arranged in lines of 6 × 6 traps. The top line of traps ran parallel to a dune crest, with the remaining lines running into the swale to ensure that the topographic variation in the environment was captured. Although cattle usually avoid dune crests, we monitored small vertebrates on all parts of the dunes because most species use them and are equally at risk of capture throughout [27]. Pitfall traps were constructed from PVC pipes (60 cm long, 15 cm diameter) that were buried flush with the ground. Traps were fitted with aluminium flywire drift fences that extended 2.5 m either side of the pits to increase the chance of intercepting and capturing surface-active animals [28], [29]. Traps were opened in the late afternoon, checked soon after dawn each day and closed on the third morning, resulting in three consecutive nights of trapping per grid per trip. Captured vertebrates were identified, weighed, measured and marked so that recaptures could be identified. Small mammals were identified using unique ear notches and lizards by removing the terminal digits of toes using sterilized surgical scissors. All animals were released at the point of capture.

Vegetation was monitored once per session concurrently with the trapping of small vertebrates. One trap per trap line was chosen at random and vegetation cover and composition were measured within a 2.5 m radius of the trap (∼20 m^2^). This produced a total of six vegetation samples from each 1-ha grid but, because initial explorations [25] revealed that cattle primarily use the dune swales, only data from the lowest three plots near the swales were used in analyses. The percentage cover of each plant species present in a plot was estimated visually [30], with plant nomenclature following the Census of the Queensland Flora (2010) [31]. By combining estimates for all vascular plant species, a value for ‘total ground cover’ was obtained. We also defined three structural groups of plants that were expected to differ in their responses to cattle grazing. These were ‘herbs and forbs’ (usually ephemeral herbs with non-woody stems or woody at the base only), ‘grasses and sedges’ (all species in the family Poaceae (except *T. basedowii*) and Cyperaceae), and ‘spinifex’ (*T. basedowii*). The first two groups provide preferred forage for cattle [32] and therefore were expected to decrease under grazing. However, as ‘herbs and forbs’ and ‘grasses and sedges’ comprise species of potentially different palatability, we also investigated separately the most common species within these groups (*Ptilotus polystachyus* and *Aristida contorta*) that have been found to decrease under grazing in previous studies [24], [26]. In contrast, the spiky leaves of spinifex hummocks (*T. basedowii*) were not expected to be palatable; hence, cover of this grass was not expected to be affected by cattle grazing during this study.

An index of reproductive output for each plant species per plot was recorded at each census. This was assessed as the product of the proportion of individual plants flowering in each plot, and the flowering intensity, averaged over all plots per grid. The index was scored on a rank scale, from 0 (flowers absent) to 5 (all individuals flowering; flowering profuse). ‘Flowers’ included clustered inflorescences such as composites, racemes and umbels. Diaspores that would be dispersed were recorded as ‘seeds’ and included fruits (e.g. pods on peas rather than the seeds within each fruit) and single seeds.

### Statistical Analyses

We used a balanced design with two factors: grazing ‘treatment’ (2 levels: ‘+ cattle’ (continuously grazed) and ‘**−** cattle’ (cattle recently removed)), and grazing ‘intensity’ (2 levels: ‘light’ and ‘heavy’ historical grazing pressure). As our data were derived from permanent grids sampled on several occasions (‘trips’) and hence were temporally non-independent, we used two-factor repeated measures ANOVAs [33], [34] for the following comparisons: total vegetation cover, cover of the three structural vegetation groups and the species *A. contorta* and *P. polystachyus*, as well as mammal and reptile abundances, the abundances of species within dominant mammal (Rodentia and Dasyuridae) and reptile families (Agamidae and Scincidae), and the abundance-index for flowers and seeds. We also explored grazing effects on individual vertebrate species with sufficient captures for analysis: the rodent *Pseudomys hermannsburgensis*, the dasyurid *Sminthopsis youngsoni*, the agamids *Ctenophorus isolepis* and *C. nuchalis*, as well as the skinks *Lerista labialis* and *Ctenotus pantherinus*. We expressed animal abundances as captures per gridnight (1 gridnight = 1 grid open for one day and night), after excluding any recaptures of individuals within the same trip to maximize independence. This catch-per-unit-effort index of abundance is robust when compared with other estimates of animal activity [27]. Our choice of trapping method was informed by long-term studies [12], [16], [30] and as mark-recapture analysis methods are not appropriate in our study system due to low recapture rates, we make the simplifying assumption of equal detection probability, at any given point in time. We do acknowledge that, as with all trapping methods, our detection of species may be affected by choice of method [35], conditions at time of survey [36], [37], interactions among species [36], [38] or by differences in abundance [36]. These issues, while important, are unlikely to have been biased in such a way that we would not be able to detect the effect of the main treatment variables of interest, namely, grazing history and recent cattle removal.

Before conducting any analyses, we examined residual plots to check for homogeneity of variances and conducted transformations if needed. Assumptions of sphericity were tested using Mauchely’s test [39] and, if significant, Greenhouse-Geisser estimates were used in the repeated measures calculations [33]. Type III sums of squares were used for ANOVAs, with all analyses performed in R. 2.10.1 [40]. We carried out all analyses using data from the balanced trips only (n = 5), in May and September 2007 and April, June and October 2008, but present data from all trips graphically to show temporal trends.

To detect changes in plant species assemblages over time, ordinations were performed on the percentage cover of plant species using non-metric multidimensional scaling (nMDS) [34]. To emphasize the effects of rare species, data were 4^th^-root transformed [33]. Bray-Curtis dissimilarity coefficients were used for the resemblance matrix and 50 iterations were used to configure minimum stress, as recommended by Anderson et al. [34]. Two-factor ANOSIMs were used to check any apparent differences in the nMDS plots. When significant, SIMPER analyses were then conducted to identify the species that contributed most to the differences between treatment groups. Because vegetation other than spinifex was either absent or so shrivelled that it could not be reliably identified prior to the heavy rains in the summer of 2006/2007, analyses of vegetation were carried out only after this rainfall event, roughly 3 months (May 2007), 6 months (September 2007), 13 months (April 2008), and 19 months (October 2008) later. Preliminary ordination analyses on small mammals and lizards suggested that too few species were captured for results to be reliable; hence, no compositional analyses are presented for these groups. Therefore, we investigated differences in vertebrate species richness and diversity using the same analyses as described above for vertebrate abundance (two-factor repeated measures ANOVA). Inspection of our sampling results suggested that several species were captured only once or twice; hence, we used the Shannon index to estimate diversity as this index accounts well for rare species [41].

## Results

Overall, our results confirm that neither vegetation nor small vertebrates necessarily responded immediately to the removal of livestock, but that rainfall events and cumulative grazing history as well as the interaction of these factors were key determinants of floral and faunal performance. Responses varied considerably across groups, as described below.

### Vegetation Cover, Reproductive Output and Species Composition

**Figure 2:**
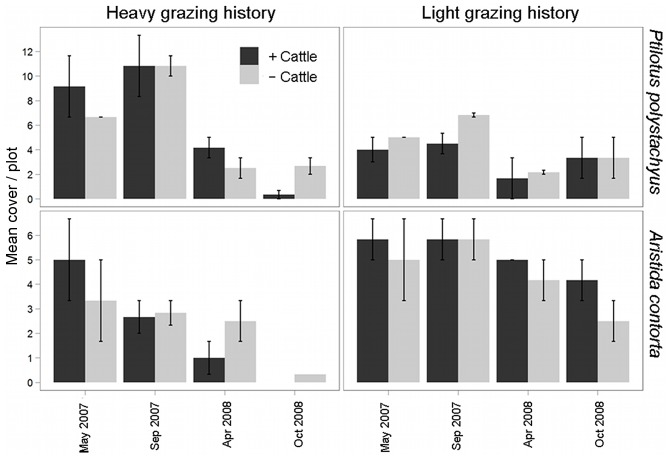
Percentage cover (mean ± SE) of the herb *Ptilotus polystachyus* and the grass *Aristida contorta*. Cover is averaged per plot, in sites with different historic grazing intensities (‘light’ and ‘heavy’) and recent cattle removal (‘+ cattle’ and ‘**−** cattle’) in the Simpson Desert, central Australia, in May 2007, September 2007, April 2008 and October 2008. Prior to heavy rains in early 2007 there was no coverage of either *P. polystachyus* or *A. contorta*; hence, data from these periods are not shown.

Of the 78 species of plants recorded across all sites (Table S1), the total number of species was higher in historically lightly compared to heavily grazed areas (64 vs 49 species, respectively). Total vegetation cover was not affected significantly by grazing intensity (Tables 1, S2). However, there was a strong trend for total cover (*F*_1,4_ = 6.499, *P* = 0.063) and spinifex cover (*F*_1,4_ = 7.464, *P* = 0.052) to be greater in areas where grazing intensity had been historically light rather than heavy. There were strong interactions between ‘trip’ (i.e. time since rain) and grazing intensity for the plant groups ‘herbs and forbs’ (*F*_1,4_ = 5.000, *P* = 0.018) and ‘grasses and sedges’(*F*_1,4_ = 9.283, *P* = 0.002) that reflected much faster rates of decline in the historically heavily grazed areas compared to slower rates of decline in the areas with light grazing history (Tables 1, S2). The most common species were affected in opposite ways, with the cover of the perennial grass *A. contorta* being greater in areas of historically light grazing compared with that in areas of heavy grazing (*F*_1,4_ = 14.269, *P* = 0.019), whereas the converse was true for the herb *P. polystachyus* (*F*_1,4_ = 13.527, *P* = 0.021) (Table 1, Fig. 2).

**Table 1:** Summary of repeated measures ANOVA results on the effects of grazing history and cattle removal on the abundance of vegetation.

	Total vegetation			Plant groups			Species		
Response		Cover		Spinifex hummocks	Grasses & sedges	Herbs & forbs		*P. poly-stachyus*	*A. contorta*
Between									
Grazing intensity		0.063		0.052				*	*
Cattle removal									
Grazing intensity × Cattle removal									
Within									
Trips		***			***	***		***	*
Trip × Grazing intensity					**	*		*	
Trip × Cattle removal									
Trip × Grazing intensity × Cattle removal						0.061			

Historic grazing intensities are ‘light’ and ‘heavy’ and cattle removal treatments are ‘+ cattle’ and ‘**−** cattle’. Common vertebrate species that deviated in their responses from those of the groups they belong to are shown, and significance levels are indicated as follows: *P<0.05, **P<0.01, ***P<0.001. Trends (*P*≤0.065) are reported as exact *P*-values. Species abbreviations: *A. contorta* = *Aristida contorta*, *P. polystachyus* = *Ptilotus polystachyus*.

**Figure 3:**
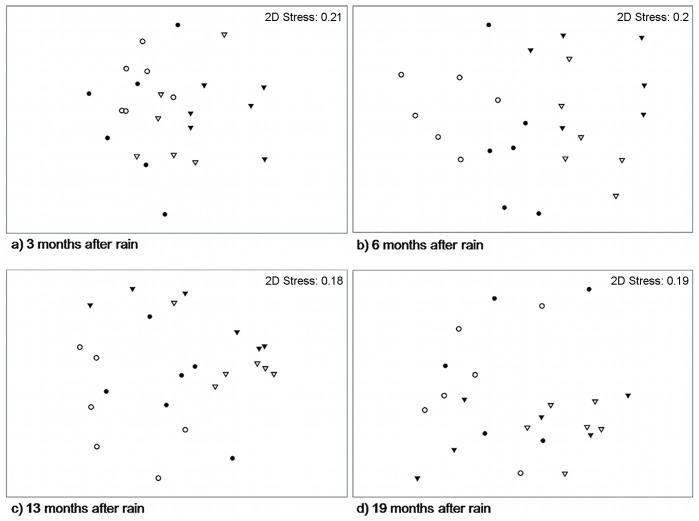
nMDS ordination plots showing differences in vegetation species assemblages under different grazing regimes. a) 3 months, b) 6 months, c) 13 months and d) 19 months after above average rainfall in early 2007. Data were fourth-root transformed and a zero-adjusted Bray-Curtis similarity matrix was used. Grazing intensity is indicated by shape (‘light’ = circle, ‘heavy’ = triangle) and grazing treatment is represented by fill shading (‘+ cattle’ = open, white), ‘**−** cattle’ = closed, black). For each of the four treatment groups there were three vegetation samples taken on each of the two replicate grids for a total of six samples per treatment.

Plant species assemblages were affected strongly by historical grazing pressure on all sampling occasions (Fig. 3), with SIMPER analyses (not shown) indicating that differences were due mainly to the consistently higher cover of *A. contorta* where grazing history had been light. Several other species also contributed to dissimilarity by their representation at different times in the historically heavily-grazed areas, with the prickly herb *Tribulus terrestris* contributing most to between-area separation with a higher cover where grazing history was heavy at 13 months after rain, and with spinifex and the perennial grass *Eragrostis eriopoda* contributing strongly at 19 months after rain with higher cover in areas with light grazing history.

The total number of plant species was similar in areas with continuous cattle grazing (58 species) and without (60 species) (Table S1). As expected, due to the short time since cattle removal, there were no effects on the cover of vegetation, but there was evidence of a reduction in reproductive output in the form of flowers where grazing history had been historically heavy (*F*_4,16_ *= *5.262, *P = *0.048, Greenhouse-Geisser adjusted). There was also some reduction of seed abundance where grazing history was heavy and an effect of time since rain that resulted in a significant three-factor interaction (*F*_3,12_ *= *2.745, *P*<0.001). In contrast to the consistent effects of the historical level of grazing across all survey dates, cattle removal affected species composition up to six months, but not 13 and 19 months, after the above average rainfall event in early 2007 (Fig. 3). Differences in species assemblages between grazing treatments at the earlier times arose from the relatively high dissimilarity (>10%) in cover of *T. basedowii*, *A. contorta* and *P. polystachyus* between treatments, and the distinct presence of several species within the Zygophyllaceae (*Tribulopis angustifolia*, *Tribulus terrestris* and *T. hystrix*) only in the ‘**−** cattle, heavy grazing’ treatment areas.

### Mammal Abundance

**Figure 4:**
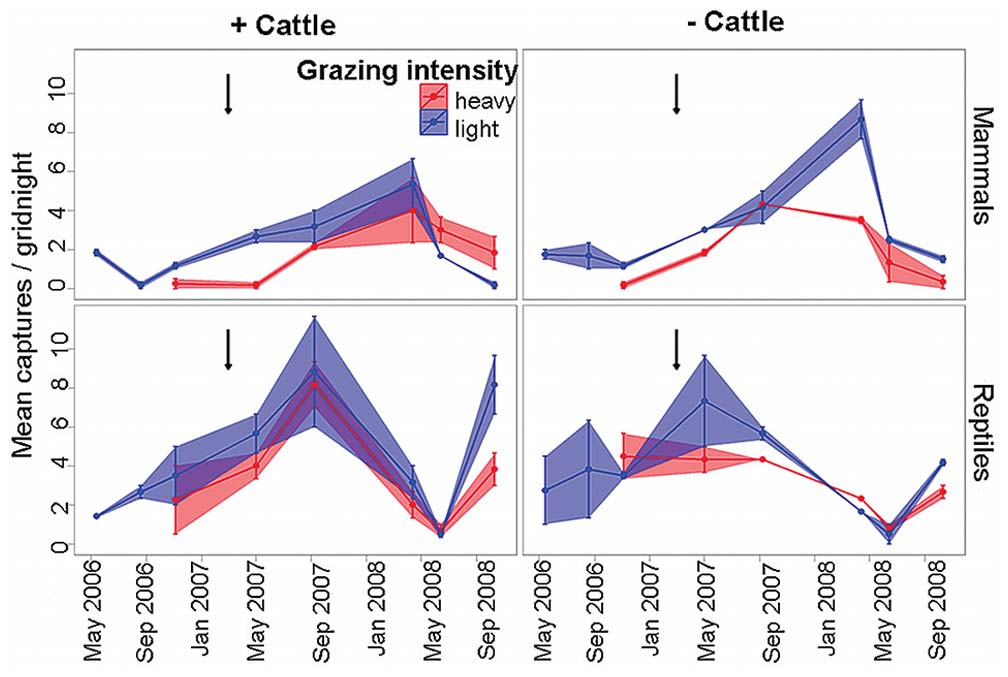
Captures (mean ± SE) of all vertebrates under different grazing histories and cattle removal treatments. Captures are averaged per gridnight and historic grazing intensities are ‘light’ and ‘heavy’ and cattle removal treatments are ‘+ cattle’ and ‘**−** cattle’. Only the trips from 2007 on, that is trips when balanced data sets were obtained (May 2007, September 2007, April 2008, June 2008 and October 2008), are used in the statistical analyses, but trips prior to 2007 are presented to give an overview of temporal trends. Arrows indicate heavy rainfall.

**Figure 5:**
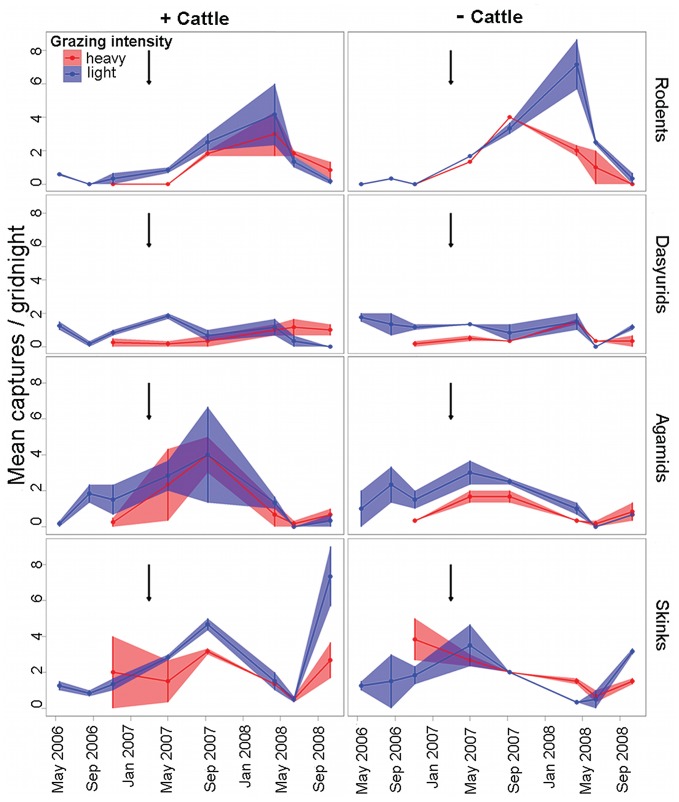
Captures (mean ± SE) of vertebrate groups under different grazing histories and cattle removal treatments. Captures are averaged per gridnight, vertebrate groups are rodents, dasyurids, agamids and skinks, historic grazing intensities are ‘light’ and ‘heavy’ and cattle removal treatments are ‘+ cattle’ and ‘**−** cattle’. Only the trips from 2007 on, that is trips when balanced data sets were obtained (May 2007, September 2007, April 2008, June 2008 and October 2008), are used in the statistical analyses, but trips prior to 2007 are presented to give an overview of temporal trends. Arrows indicate heavy rainfall.

Over the study period 506 mammals of eight species were caught, including 13% recaptures. Abundance was generally higher in areas with historically light grazing history (Fig. 4, 5). After peak abundance was reached in the ‘− cattle – light grazing’ treatment in April 2008, capture rates fell substantially in the lightly grazed areas producing a strong interaction of time since rain (‘trip’) and grazing intensity (Tables 2, S3a). There was no effect of cattle removal alone on the abundance of small mammals (*F*_3,12_ *= *4.345, *P = *0.105), but there was a significant three-way interaction of time since rain (‘trip’), treatment and intensity (*F*_3,12_ *= *3.206, *P = *0.041) that was driven by rodents increasing much more strongly about 6 months after rain where cattle had been recently removed in the lightly grazed areas (Fig. 5).

**Table 2:** Summary of repeated measures ANOVA results on the effects of grazing history and cattle removal on the abundance of small vertebrates.

	Mammals				Reptiles			
Response	Total	Rodents	Dasyurids	Diversity	Total	Agamids	Skinks	Diversity
Between								
Grazing intensity	*	*					0.064 (0.054 *L. labialis*)	
Cattle removal							(** *C. pantherinus*)	
Grazing intensity ×Cattle removal								
Within								
Trips	***	***	**	*	***	**	***	***
Trip × Grazing intensity	**	**	**				(*** *L. labialis*; ** *C. pantherinus*)	
Trip × Cattle removal					*	(0.061 *C. isolepis*)	**	
Trip × Grazing intensity× Cattle removal	*	*	*	0.055			(* *L. labialis*; 0.059 *C. pantherinus*)	

Historic grazing intensities are ‘light’ and ‘heavy’ and cattle removal treatments are ‘+ cattle’ and ‘**−** cattle’. Common vertebrate species that deviated in their responses from those of the groups they belong to are shown, and significance levels are indicated as follows: *P<0.05, **P<0.01, ***P<0.001. Trends (*P*≤0.065) are reported as exact *P*-values. Species abbreviations: *C. pantherinus* = *Ctenotus pantherinus*, *L. labialis* = *Lerista labialis*, *C. isolepis = Ctenophorus isolepis*.

Small mammals in this study comprised three species of rodents (71% of captures) and five species of dasyurids (29% of captures) (Table 3). Because *P. hermannsburgensis* dominated so strongly among the rodents and *S. youngsoni* among the dasyurids, the patterns of abundance of these species and the groups they belong to were almost identical in all treatment areas (compare Tables S4a and S4b). In the interpretation of results below, we therefore focus on the family-level patterns and note the few differences for individual species where they occur. Rodents were usually more abundant where grazing had been historically light compared to where it had been heavy, but this switched at the end of the study (from about 14 months after rain) in areas where cattle still grazed, resulting in significant interactions between time since rain (‘trip’) and grazing intensity and an interaction of all three factors (Fig. 5; Tables 2, S4a). Similar to the rodent response, dasyurid abundance was affected by the interaction of the historical intensity of grazing and time since rain, as well as by the interaction of all three factors (Fig. 5; Tables 2, S4a). There were insufficient captures of any other species for repeated measures analysis but, except for *Ningaui ridei* (n = 6), all the remaining species’ total captures were more than twice as high where grazing history was light compared to where it was heavy (Table 3).

**Table 3:** Total captures of small mammals under different grazing histories and cattle removal treatments.

			Heavy grazing		Light grazing		
	Species		+ cattle	− cattle	+ cattle	− cattle	TOTAL
Dasyurids	*Dasycercus blythi*		2	0	3	2	7
	*Ningaui ridei*		1	2	2	1	6 (16)
	*Sminthopsis hirtipes*		3	1	5	5	14 (21)
	*Sminthopsis macroura*		0	2	0	0	2
	*Sminthopsis youngsoni*		16	13	14	21	64 (87)
Rodents	*Mus musculus*		2	2	0	3	7 (8)
	*Notomys alexis*		2	5	7	10	24 (41)
	*Pseudomys hermannsburgensis*		41	43	47	77	208 (289)
	**TOTAL**		67	68	78	119	332 (471)

Historic grazing intensities are ‘light’ and ‘heavy’ and cattle removal treatments are ‘+ cattle’ and ‘**−** cattle’. Numbers in brackets are total captures over all eight trips, if different from those captured during trips (May and September 2007 and April, June and October 2008) when balanced datasets were obtained. Recaptures within trips have been excluded.

In summary, capture rates of small mammals generally increased after the heavy rainfall event in early 2007 before collapsing to low levels in 2008. They were affected significantly by grazing intensity and time since rain (‘trip’) and the interaction of these factors but not, with the exception of its contribution in the time since rain (‘trip’) × grazing intensity × treatment interaction, by recent cattle removal (Tables 2, S3a; Fig. 4). However, most species that were caught too infrequently for analysis (five out of eight) achieved twice as many captures where cattle had been recently removed compared to where cattle still grazed (Table 3).

### Reptile Abundance

Reptiles as a group (693 captures, 2% recaptures, 33 species) were not affected by grazing history (Fig. 4, Table S5), but different species responded differently to grazing conditions, as we describe below. However, there was a significant interaction between time since rain (‘trip’) and grazing treatment (*F*_3,12_ *= *4.696, *P = *0.011) for reptiles as a group (Tables 2, S3b). This arose largely because reptile abundance reached its peak two months after rain (May 2007) in areas where cattle had been recently removed and six months after rain (September 2007) where cattle still grazed (Fig. 4). In late spring at about 18 months after rain (October 2008), reptile abundance increased and was higher in light and heavily grazed areas where cattle still grazed than where cattle had been recently removed (Fig. 4). High variances in reptilian capture rates precluded the detection of any further significant treatment differences, with different species apparently responding differently to grazing conditions. Sources of this variation are considered further below.

Four species accounted for 91% of all reptile captures (Table S5). Over half of all captures comprised species within the family Scincidae (52%) and more than a third of all captures represented the family Agamidae (39%). The most dominant skink was *Lerista labialis* which accounted for over half of all captures of skinks (53%) and a fourth of all reptiles captured (25%). The second most abundant skink and fourth most abundant reptile species (10%) was *Ctenotus pantherinus*. Agamids were dominated by *Ctenophorus isolepis* and *Ctenophorus nuchalis*, each accounting for 19% of all reptile captures. Other species (n = 29) individually accounted for less than 5% of reptile captures and were unsuitable for repeated measures analyses (Table S5).

While agamids were not affected by grazing history (*F*_3,12_ *= *0.306, *P = *0.609), skinks were slightly affected (*F*_3,12_ *= *6.457, *P = *0.064) and showed a significant interaction between grazing history and time since rain (‘trip’) (*F*_3,12_ *= *6.354, *P = *0.001) (Fig. 5; Tables 2, S6a); very similar patterns were observed for the most abundant species within both the Agamidae and Scincidae (Table S6b). The skink *C. pantherinus* reached peak abundance 6 months after rain (September 2007) where cattle still grazed and where historical grazing pressure was low, resulting not only in a significant time since rain (‘trip’) and grazing treatment interaction (*F_3_*_,12_ *= *4.069, *P = *0.018), but also in a main effect of grazing treatment (*F*_3,12_ *= *26.270, *P = *0.007) (Tables 2, S6b).

In summary, reptiles as a group responded earlier to rainfall than mammals. They also responded less clearly due to contrasting responses at lower taxonomic levels that could only be investigated statistically for the four most common species.

### Small Mammal and Reptile Species Richness and Diversity

Mammal and reptile species richness and diversity fluctuated markedly over time. Neither richness nor diversity responded to historical grazing intensity or current grazing treatment in any consistent manner (Tables 2, S7), although mammal species richness was influenced by the interaction of time since rain (‘trip’), historical grazing intensity and current grazing treatment (*F*_4,16_ = 3.531, *P* = 0.030).

## Discussion

Our results confirm that neither vegetation nor small vertebrates necessarily respond immediately to the removal of livestock, but that rainfall events and cumulative grazing history are key determinants of floral and faunal performance in the arid grassland landscapes we studied. Some species of plants and small vertebrates could be identified as ‘decreasers’ in areas that had experienced heavy historical grazing, and there was a strong compositional difference in plant species between areas with different histories of grazing pressure. Recent cattle removal alone produced few clear effects, but there was evidence of a negative impact of continuous grazing on plant species composition and the abundance of flowers and seeds at certain times after rain. This impact was particularly obvious in the more heavily grazed eastern parts of the study area than the west, even though rainfall during the period of study was slightly greater in the east and thus could have been expected to more strongly ameliorate grazing impacts there. Typically, vegetation cover and the abundance, species richness and diversity of small vertebrates changed markedly over the course of the study, all responding strongly to the rainfall event in 2007. We discuss the differing effects of the historical grazing intensity, recent cattle removal and time since rainfall below.

### Effects of Different Grazing History

#### Vegetation

Despite the relatively recent cattle grazing history in the study region, which commenced only in the 1970s [42], cattle appear already to have altered vegetation composition in areas where they have grazed more heavily. Among the more prominent species contributing to compositional differences between areas of different grazing history were members of the Zygophyllaceae (*Tribulopis angustifolia*, *Tribulus hystrix* and *T. terrestris*). Some species in these genera have been classified as unpalatable to stock [43] and, in this study, we found no clipped leaves or other evidence of grazing upon them. The prostrate growth form of *Tribulus* spp., and *T. angustifolia*, contributed importantly to total ground cover, especially in areas where grazing has been heavy. Negative effects of heavy historic grazing were observed for the palatable grass *Aristida contorta*. This species also has been identified as a ‘decreaser’ under heavy grazing pressure in the study system [24], [26], [42], and we saw much evidence in the present work that it had been cropped by grazers.

The herb *Ptilotus polystachyus* appeared initially to be an ‘increaser’ species in areas with a history of heavy grazing pressure, achieving greater levels of cover where grazing had been heavy rather than light three months after rain in 2007. However, this species may be susceptible to heavy continued grazing pressure; its cover declined sharply between April and October 2008 only in cattle-present areas at a time when not much other food was available for cattle. Other authors also have identified this species as a ‘decreaser’ under heavy grazing [24], [26], [42]. The increased cover in areas of light grazing history between April and October 2008 is puzzling, but could possibly be attributed to many *P. polystachyus* plants growing within the centre of spinifex hummocks where moisture might have been trapped, or to a patchy local rainfall event that fell onsite but missed our rain gauge slightly further to the west of the light grazing history sites. There was, however, little other evidence of a response by other vegetation.

Total vegetation cover showed a tendency to remain higher in areas where grazing intensity had been historically light. The lack of a strong historic grazing effect on vegetation cover was likely due to the change in composition, with some plant species responding positively to heavy grazing and others responding negatively, thus resulting in a ‘cancelling’ effect when total cover was considered.

#### Small vertebrates

Past grazing pressure had a pronounced effect on the abundance of small mammals, with populations achieving higher abundances where grazing intensity had been historically light rather than heavy, although this depended also on the time of sampling and on recent cattle removal. Small mammals may have responded positively to the higher levels of spinifex cover in the lightly**-** compared to heavily-grazed areas, as spinifex is crucial in providing shelter from predation [44]–[46]. The sharper drop in rodent abundance where grazing intensity was light, compared to where it was heavy, in June and October 2008, could have arisen due to the follow up rains that were lower in the historically lightly grazed west (11 mm) compared the heavily grazed east (65 mm). Another reason could be elevated predation as areas with more prey could attract more predators. Both red foxes *Vulpes vulpes* and feral cats *Felis catus* show delayed numerical responses to increases in small mammal populations, typically peaking some 12–18 months after above-average rainfall events in the study system [13].

Patterns in the abundance of rodents were driven largely by the dominant native mouse *Pseudomys hermannsburgensis*. Capture rates of the rodents increased dramatically in the wake of the heavy rains in early 2007, with populations responding most probably via elevated reproduction and rapid recruitment under the favourable conditions [47], [48].

Although less abundant, dasyurid marsupials responded to grazing conditions in a similar manner to the rodents, with the dominant *Sminthopsis youngsoni* in particular achieving higher numbers where grazing history had been light compared to where it was heavy. As *S. youngsoni* is a carnivorous food generalist [49] that appears to be largely unaffected by higher predation risk in open habitat [50], we had not expected this species to be affected strongly by grazing. However, as it forages preferentially near spinifex hummocks [51], it may have benefited from the less-trampled spinifex cover in the lightly grazed areas rather than from differences in the grazing regime *per se*. Other species of dasyurids have been shown to be tolerant of grazing [52] or to decline only in heavily grazed sites [53].

Responses by reptiles to grazing were variable. Agamids showed no indication of a response to different historical grazing pressure. The presence of spinifex generally is important for reptiles [54], [55], and this is true for both species of *Ctenophorus* studied here [56]. The effects of cattle grazing on reptiles can be ameliorated where grazing is not heavy enough to substantially reduce spinifex cover [55], and this may be the case for these agamids.

In contrast to the agamids, the two most abundant species of skinks were affected by differences in grazing history at certain times. During periods of peak capture, numbers of *Ctenotus pantherinus* were highest where grazing has been light (‘+ cattle – light’), but capture rates interacted also with grazing history and time. This skink is generally seen as a decreaser species under livestock grazing [57], but our study suggests that it can tolerate light grazing, especially if moderate levels of vegetation are available for shelter [58]. Termites form the bulk of the diet, and prolonged heavy grazing likely depresses this food source. For the most abundant skink, *Lerista labialis* [59], captures varied over time and patterns of response to grazing history and treatment were inconsistent. As this species is largely subterranean and prefers the loose sand on dune crests [59], it was not expected to be impacted greatly by cattle as cattle seldom use dune crests [20]. These conclusions indicate that the two most abundant skinks in the study persist under light grazing conditions, and accord also with the prevailing view that most species decline where grazing is heavy [53], [57].

The inconsistent patterns in small mammal species richness and diversity to different grazing histories probably reflect the small number of species in the system and low numbers of captures of six of the eight species that were present. Low numbers and highly variable capture rates are typical of small mammals in arid Australia [29], [60], [61]. This makes it generally difficult to detect strong signals in response to different grazing pressures [62], especially if the response metrics depend on reliably capturing the suite of species present. As with small mammals, trip had the strongest influence on the richness and diversity of reptiles. Inspection of the raw data indicated that some reptiles, such as geckoes and pygopodids, were captured only in summer, thus contributing to the time effect. In addition, 13 of the 33 species captured were represented by just one or two individuals. The sporadic nature of these records suggests that some species were genuinely rare or that our capture techniques were not equally efficient for all taxa. Irrespective, the paucity of captures of so many species makes it difficult to draw reliable conclusions about how reptile diversity may respond to differences in historic grazing intensity.

### Effects of Cattle Removal

#### Vegetation

Evidence for positive effects of recent cattle removal was found for plant species composition when vegetation cover and plant species richness were highest at about 3–6 months after rain and for flowers and seeds about a year after rain where grazing activity was heavy rather than light. Considering that unpalatable plants usually remain established even after the removal of herbivores (e.g. Seymour et al. [63]), it is not surprising that we did not find a change in total vegetation cover after cattle removal.In addition to whether plant species are palatable to livestock, there are other, more subtle, explanations that may help to account for the stronger effects of grazing intensity than recent cattle removal. Firstly, as there was a tendency for areas with a history of light grazing to have greater spinifex cover than those with a history of heavy grazing, the spinifex itself may have afforded palatable plant species some protection. Grasses such as *Eragrostis setifolia* and herbs and small shrubs such as *Blennodia canescens* and *Enchylaena tomentosa* frequently grow near or even through spinifex hummocks, and may experience lower grazing pressure there. These species were found only in areas with a history of light grazing. Secondly, soils in heavily grazed areas may be more compacted and have lower levels of nutrients than soils in lightly grazed areas, so that ephemeral plants on these soils achieved low cover and declined rapidly when stressed by the onset of dry conditions. It is unlikely that the patterns of vegetation response we observed were driven by local differences in rainfall history [64]. The possibilities that cattle were not effectively removed or that other large grazing mammals dispersed in to replace cattle as they were removed also can be rejected, as field surveys revealed no cattle sightings at our ‘**−** cattle’ sites from October 2006 on, and also showed no compensatory increases in kangaroos or camels throughout the study [25].

#### Small vertebrates

Recent cattle removal alone produced no clear effects on small vertebrates within the study period. This finding contrasts with the results of studies in the tropical savannas of Australia [65], [66], but could be due to generally heavier stocking in the savannas compared to the desert systems. It is possible that insufficient time had elapsed for any benefits of cattle removal to be manifest, especially if such benefits are mediated through vegetation structure and as the rains in 2007 stimulated an increase in primary productivity across the landscape. In South Australia, for example, Read and Cunningham [53] recorded fewer rodents in heavily compared to moderately and lightly cattle-grazed sites, and highest numbers inside a cattle-free exclosure, but effects were manifest only after a decade. Effects of grazing may depend also on the scale of sampling [67], with more rapid small mammal responses likely to be observed in small, fenced exclosures that keep out cattle and all other large mammals (e.g. Keesing and Crawford [68]) than in the very large scale cattle removal studied here.

The highest capture rates of the most abundant lizards occurred where cattle still grazed; for agamids this was in September 2007 and for skinks it was in October 2008. This temporal shift could reflect differences in family-level preferences for different temperatures or other micro-climatic conditions that prevailed during the respective sampling periods [69], or responses to other, more subtle, environmental cues. Whatever the reason, it is hard to attribute the pattern to differential responses to grazing conditions. As reptiles are often considered resilient to grazing impacts [53], [70], it can be difficult to detect differences between different grazing treatments if captures are dominated by a few species that show neutral responses to grazing [71]. Common species often are habitat generalists that are likely to be less affected by disturbances such as grazing [54]. Agamids, like *C. nuchalis*, often exploit open areas, including freshly burnt sites [61], [72], and can increase under livestock grazing [53], [57]. We found capture rates of *C. nuchalis* and *C. isolepis* to be slightly higher in the half year following rain where cattle were present compared to where cattle had been recently removed but, due to high spatial variability in captures, there was no clear pattern of them being more associated with continuously cattle-grazed than recently cattle-free areas.

The marked rise in skink abundance in the ‘+cattle’ sites in late 2008 may have arisen in part from *L. labialis* exploiting sand that had been disturbed by cattle, as this species prefers loose sand for burrowing [59]. The abundance of *Ctenotus pantherinus* was more often higher where cattle grazed compared to where cattle had been recently removed. Although this skink is generally seen as a decreaser species under livestock grazing [57], [73], it clearly persists where grazing pressure is light and could even benefit if it is able to exploit termites and other invertebrates that colonize the dry dung of livestock [58].

There was no clear effect of recent cattle removal on species richness and diversity of small vertebrates. The time since removal may have been too short to have allowed any species that had experienced local extinction under grazing to immigrate back into the now-cattle free areas.

### Implications

This study shows that, at least in the arid grassland system we studied, it is important to consider the cumulative effects of grazing because current grazing pressure alone, particularly when compared to recent cattle removal, does not provide a reliable indicator of impact on the biota. The effects of recent grazing removal were strongly dependent on historical grazing intensity. If such low rainfall systems are to retain their resilience and the diversity of their native species, we suggest that it would be beneficial to introduce more oversight into livestock production. For example, if annual returns were required on the stocking rates of domestic animals and the abundance of feral herbivores, on the paddocks or other locations where grazing was allowed, and on the timing of grazing in relation to environmental conditions such as drought or rain, actual grazing pressure over time could be quantified and open for audit on the public record. Such record keeping is standard in other production industries such as fisheries and kangaroo harvesting, as it is for feral herbivores on stock-free conservation areas [74]. Stocking records would also allow evaluation of whether ‘light’ grazing pressure could be best achieved by continuous low stocking or by short periods of heavy grazing during favourable times after rain combined with radical destocking at the beginning of drought. The benefits of other practices such as rotational grazing, which is used widely in some dryland regions of the world [75] and currently on Carlo station in the present study, could also be quantified if stocking records were to be kept and made available.

Our study showed further that total vegetation cover is a poor indicator of grazing impact, as unpalatable plant species can replace palatable ones. This suggests that palatable plant species such as *Aristida contorta* could be used as target species for destocking decisions and their increase as a sign of recovery from grazing. As we found that abundances of flowers and seeds were often reduced where cattle grazing occurred, monitoring the reproductive output of palatable species could also aid in stock management.

Unfortunately, there was little indication that palatable plants could act as surrogates for all other taxa; species and families of vascular plants and vertebrates responded differently to grazing. That the responses of small vertebrates failed to show any clear responses could be because captures of vertebrates were highly variable, for most species scant and driven by a few dominant generalist species which may not be affected by disturbance such as continuous grazing as much as rarer more specialised species. Using methods in future studies which account for imperfect detection in multispecies systems, for example using occupancy modelling [76], [77] or estimating abundance of cryptic species using trapping point transects in mark-recapture sampling [78] could help in detecting grazing effects more reliably in rarer species. However, as many taxa can take years or even decades to recover from the impacts of cattle grazing [e.g. 79,80,81], long term studies are likely to be needed to track their recovery. In arid systems recovery from disturbance such as grazing relies strongly on rainfall [82]. Hence, monitoring of diverse biotic responses to grazing and recovery from grazing might be carried out most profitably in years following heavy rainfall events.

## Supporting Information

Table S1 **Presence/absence of plant species accumulated over the 3 year study period in sites with different historic grazing intensities (‘light’ and ‘heavy’) and recent cattle removal (‘+ cattle’ and ‘− cattle’) in the Simpson Desert, central Australia.** Species are ordered by family, x = present and no symbol = not detected.
(DOCX)

Table S2 **Repeated measures ANOVA results on the effects of historic grazing intensity (‘light’ and ‘heavy’) and recent cattle removal (‘+ cattle’ and ‘− cattle’) in the Simpson Desert, central Australia, on cover of ‘total vegetation’, ‘spinifex hummocks’, ‘herbs & forbs’ and ‘grasses & sedges’.** Significant results (*P*<0.05) are shown in bold.
(DOCX)

Table S3 **Repeated measures ANOVA results on the effects of historic grazing intensity (‘light’ and ‘heavy’) and recent cattle removal (‘+ cattle’ and ‘**−** cattle’) in the Simpson Desert, central Australia, on average abundances of a) small mammals and b) reptiles.** MS = mean square, SS = sums of squares, df = degrees of freedom. Significant results (*P*<0.05) are shown in bold.
(DOCX)

Table S4 **Repeated measures ANOVA results on the effects of historic grazing intensity (‘light’ and ‘heavy’) and recent cattle removal (‘+ cattle’ and ‘**−** cattle’) in the Simpson Desert, central Australia, on average abundances of a) Rodentia and Dasyuridae and b) *Pseudomys hermannsburgensis* and *Sminthopsis youngsoni*.** Degrees of freedom for between factor tests were 1, 4 and for within factors 4, 16, for all analyses. Significant results (*P*<0.05) are shown in bold.
(DOCX)

Table S5 **Total captures of reptiles in sites with different historic grazing intensities (‘light’ and ‘heavy’) and recent cattle removal (‘+ cattle’ and ‘−** c**attle’) in the Simpson Desert, central Australia.** Numbers in brackets are total captures over all trips, if different from those captured during trips when balanced datasets were obtained. Recaptures within trips have been excluded.
(DOCX)

Table S6 **Repeated measures ANOVA results on the effects of historic grazing intensity (‘light’ and ‘heavy’) and recent cattle removal (‘+ cattle’ and ‘**−** cattle’) in the Simpson Desert, central Australia, on average abundances of a) Agamidae and Scincidae and b) *Ctenophorus isolepis, C. nuchalis, Lerista labialis* and *Ctenotus pantherinus*.** Degrees of freedom for between factor tests were 1, 4 and for within factors 4, 16, if not stated otherwise. Significant results (*P*<0.05) are shown in bold.
(DOCX)

Table S7 **Repeated measures ANOVA results on the effects of historic grazing intensity (‘light’ and ‘heavy’) and recent cattle removal (‘+ cattle’ and ‘**−** cattle’) in the Simpson Desert, central Australia, comparing the average species richness and diversity per grid and trip in all four treatment combinations, for a) small mammals and b) reptiles.** Degrees of freedom for between factor tests were 1, 4 and for within factors 4, 16, if not stated otherwise. Significant results (*P*<0.05) are shown in bold.
(DOCX)
