# Supplementary material for: Interactions of Grazing History, Cattle Removal and Time since Rain Drive Divergent Short-Term Responses by Desert Biota
Source: PLoS One. 2013 Jul 16;8(7):e68466. doi: 10.1371/journal.pone.0068466 (PMC3713037; doi:10.1371/journal.pone.0068466)
Supplement: Table S1 — Presence/absence of plant species accumulated over the 3 year study period in sites with different historic grazing intensities (‘light’ and ‘heavy’) and recent cattle removal (‘+ cattle’ and ‘− cattle’) in the Simpson Desert, central Australia. Species are ordered by family, x = present and no symbol = not detected. (DOCX) [file pone.0068466.s001.docx]

**Table S1**. Presence / absence of plant species accumulated over the 3 year study period in sites with different historic grazing intensities (‘light’ and ‘heavy’) and recent cattle removal (‘+ cattle’ and ‘- cattle’) in the Simpson Desert, central Australia. Species are ordered by family, x = present and no symbol = not detected.

|  |  | **Heavy grazing** | | **Light grazing** | |  |  | **Heavy grazing** | | **Light grazing** | |
| --- | --- | --- | --- | --- | --- | --- | --- | --- | --- | --- | --- |
| **Family** | **Species** | **+ cattle** | **- cattle** | **+ cattle** | **- cattle** | **Family** | **Species** | **+ cattle** | **- cattle** | **+ cattle** | **- cattle** |
| Aizoaceae | *Trianthema pilosa* F.Muell. |  | x |  |  | Malvaceae | *Abutilon otocarpum* F.Muell. |  | x |  | x |
| Amaranthaceae | *Ptilotus latifolius* R.Br. | x |  | x |  |  | *Keraudrenia* sp. |  |  |  | x |
|  | *Ptilotus polystachyus* (Gaudich.) F.Muell. | x | x | x | x |  | *Keraudrenia nephrosperma* Benth. |  |  |  | x |
| Araliaceae | *Trachymene glaucifolia* (F.Muell.) Benth. | x | x | x | x |  | *Malvastrum americanum* (L.) Torr. |  | x |  |  |
| Asteraceae | *Calocephalus platycephalus* (F.Muell.) Benth. |  | x |  |  |  | *Sida* spp. | x | x | x | x |
|  | *Calotis erinacea* Steetz |  |  | x | x | Meliaceae | *Owenia acidula* F.Muell. | x |  |  |  |
| Boraginaceae | *Halgania cyanea* Lindl. |  |  | x |  | Mimosaceae | *Acacia bivenosa* DC. |  |  |  | x |
|  | *Trichodesma zeylanicum* (Burm.f.) R.Br. | x |  | x | x |  | *Acacia ligulata* A.Cunn. ex Benth. | x |  | x |  |
| Brassicaceae | *Blennodia canescens* R.Br. |  |  |  | x |  | *Acacia stenophylla* A.Cunn. ex Benth. |  |  | x |  |
|  | *Lepidium phlebopetalum* (F.Muell.) F.Muell. |  |  | x | x |  | *Acacia stipuligera* F.Muell. |  |  |  | x |
| Brunoniaceae | *Brunonia australis* Sm. Ex R.Br. |  |  | x | x | Myoporaceae | *Eremophila macdonnellii* F.Muell. |  |  | x |  |
|  |  | **Heavy grazing** | | **Light grazing** | |  |  | **Heavy grazing** | | **Light grazing** | |
| **Family** | **Species** | **+ cattle** | **- cattle** | **+ cattle** | **- cattle** | **Family** | **Species** | **+ cattle** | **- cattle** | **+ cattle** | **- cattle** |
| Caesalpiniaceae | *Petalostylis cassioides* (F.Muell.) Symon |  |  | x |  | Myrtaceae | *Eucalyptus pachyphylla* F.Muell. |  |  |  | x |
|  | *Senna artemisoides* subsp*. oligophylla* (F.Muell) Randell |  |  | x | x |  | *Corymbia terminalis* (F.Muell.) K.D.Hill & L.A.S.Johnson |  |  |  | x |
|  | *Senna pleurocarpa* (F.Muell.) Randell |  | x | x | x | Poaceae | *Aristida contorta* F.Muell. | x | x | x | x |
| Chenopodiaceae | *Enchylaena* tomentosa R.Br. |  |  |  | x |  | *Dactyloctenium radulans* (R.Br.) P.Beauv. |  | x |  |  |
|  | *Salsola* kali L. |  |  |  | x |  | *Eragrostis eriopoda* Benth. | x | x | x | x |
| Cleomaceae | *Cleome* viscosa L. | x | x |  |  |  | *Eragrostis setifolia* Nees |  |  |  | x |
| Convolvulaceae | *Convolvulus erubescens* Sims | x | x | x | x |  | *Eragrostis sp.* |  |  | x |  |
| Cucurbitaceae | *Cucumis maderaspatanus* L. |  |  |  | x |  | *Eriachne aristidea* F.Muell. | x | x | x | x |
| Cyperaceae | *Fimbristylis* sp. |  |  |  | x |  | *Plagiosetum refractum* (F.Muell.) Benth. | x | x | x | x |
| Euphorbiaceae | *Euphorbia drummondii* Boiss. | x | x | x | x |  | *Tragus australianus* S.T.Blake | x |  |  | x |
|  | *Euphorbia tannensis* Spreng. | x | x | x | x |  | *Triodia basedowii* E.Pritz. | x | x | x | x |
| Fabaceae | *Crotalaria cunninghamii* R.Br. | x | x | x | x |  | *Yakirra australiensis* (Domin) Lazarides & R.D.Webster | x | x | x | x |
|  | *Crotalaria eremaea* F.Muell. | x | x | x | x | Portulacaceae | *Calandrinia balonensis* Lindl. | x | x |  |  |
|  | *Cullen australasicum* (Schltdl.) J.W.Grimes. | x |  | x |  |  | *Portulaca intraterranea* J.M.Black | x |  |  |  |
|  | *Indigofera linifolia* (L.f.) Retz. |  |  | x |  |  | *Portulaca oleracea* L. | x | x |  |  |
|  |  | **Heavy grazing** | | **Light grazing** | |  |  | **Heavy grazing** | | **Light grazing** | |
| **Family** | **Species** | **+ cattle** | **- cattle** | **+ cattle** | **- cattle** | **Family** | **Species** | **+ cattle** | **- cattle** | **+ cattle** | **- cattle** |
| Fabaceae | *Swainsona microphylla* A.Gray |  |  | x |  | Proteaceae | *Grevillea juncifolia* Hook. |  |  | x |  |
|  | *Tephrosia rosea* F.Muell. ex Benth. | x | x | x | x |  | *Grevillea stenobotrya* F.Muell. | x | x | x | x |
| Goodeniaceae | *Goodenia cycloptera* R.Br. | x | x | x | x |  | *Grevillea* sp. |  |  | x |  |
|  | *Goodenia* sp. |  | x | x |  |  | *Grevillea striata* R.Br. | x | x | x |  |
|  | *Lechenaultia divaricata* F.Muell. |  | x |  | x | Rubiaceae | *Oldenlandia pterospora* (F.Muell.) F.Muell. |  | x | x | x |
|  | *Scaevola depauperata* R.Br. | x |  | x | x |  | *Spermacoce auriculata* F.Muell. |  |  | x | x |
|  | *Scaevola parvibarbata* Carolin |  |  | x |  | Sapindaceae | *Alectryon oleifolius* (Desf.) S.T.Reynolds | x |  |  |  |
|  | *Scaevola parvifolia* F.Muell. ex Benth. |  |  | x | x |  | *Atalaya hemiglauca* (F.Muell.) F.Muell. ex Benth. | x |  |  |  |
|  | *Velleia connata* F.Muell. |  |  | x |  | Sterculiaceae | *Rulingia loxophylla* F.Muell. | x | x | x | x |
| Haloragaceae | *Haloragis gossei* F.Muell. | x |  | x | x | Tiliaceae | *Triumfetta winneckeana* F.Muell. |  | x |  | x |
| Lamiaceae | *Dicrastylis costelloi* F.M.Bailey | x | x | x |  | Zygophyllaceae | *Tribulopis angustifolia* R.Br. | x | x |  |  |
|  | *Newcastelia cephalantha* F.Muell. |  | x | x |  |  | *Tribulus cistoides* L. |  | x |  |  |
|  | *Newcastelia spodiotricha* F.Muell. | x |  | x | x |  | *Tribulus terrestris* L. | x | x |  |  |
