# Supplementary material for: Interactions of Grazing History, Cattle Removal and Time since Rain Drive Divergent Short-Term Responses by Desert Biota
Source: PLoS One. 2013 Jul 16;8(7):e68466. doi: 10.1371/journal.pone.0068466 (PMC3713037; doi:10.1371/journal.pone.0068466)
Supplement: Table S2 — Repeated measures ANOVA results on the effects of historic grazing intensity (‘light’ and ‘heavy’) and recent cattle removal (‘+ cattle’ and ‘− cattle’) in the Simpson Desert, central Australia, on cover of ‘total vegetation’, ‘spinifex hummocks’, ‘herbs & forbs’ and ‘grasses & sedges’. Significant results (P<0.05) are shown in bold. (DOCX) [file pone.0068466.s002.docx]

**Table S2**. Repeated measures ANOVA results on the effects of historic grazing intensity (‘light’ and ‘heavy’) and recent cattle removal (‘+ cattle’ and ‘- cattle’) in the Simpson Desert, central Australia, on cover of ‘total vegetation’, ‘spinifex hummocks’, ‘herbs & forbs’ and ‘grasses & sedges’. Significant results (*P* < 0.05) are shown in bold.

| **Vegetation cover** | **Total** | | **Spinifex** | | **Herbs & forbs** | | **Grasses & sedges** | |
| --- | --- | --- | --- | --- | --- | --- | --- | --- |
| Source | *F* | *P* | *F* | *P* | *F* | *P* | *F* | *P* |
| Between |  |  |  |  |  |  |  |  |
| Grazing intensity | 6.499 | 0.063 | 7.464 | 0.052 | 0.048 | 0.838 | 4.908 | 0.091 |
| Grazing treatment | 0.435 | 0.546 | 5.125 | 0.086 | 0.048 | 0.838 | 1.767 | 0.255 |
| Grazing intensity x Cattle removal | 0.634 | 0.470 | 1.030 | 0.368 | 0.048 | 0.838 | 1.485 | 0.290 |
| Within |  |  |  |  |  |  |  |  |
| Trips | 30.123 | **<0.001** | 1.218 | 0.345 | 18.788 | **<0.001** | 22.981 | **<0.001** |
| Trip x Grazing intensity | 2.920 | 0.078 | 1.810 | 0.199 | 5.000 | **0.018** | 9.283 | **0.002** |
| Trip x Cattle removal | 1.291 | 0.322 | 1.989 | 0.169 | 1.000 | 0.426 | 0.981 | 0.434 |
| Trip x Grazing intensity x Cattle removal | 0.204 | 0.892 | 0.194 | 0.898 | 3.222 | 0.061 | 0.792 | 0.521 |
