# Supplementary material for: Interactions of Grazing History, Cattle Removal and Time since Rain Drive Divergent Short-Term Responses by Desert Biota
Source: PLoS One. 2013 Jul 16;8(7):e68466. doi: 10.1371/journal.pone.0068466 (PMC3713037; doi:10.1371/journal.pone.0068466)
Supplement: Table S3 — Repeated measures ANOVA results on the effects of historic grazing intensity (‘light’ and ‘heavy’) and recent cattle removal (‘+ cattle’ and ‘− cattle’) in the Simpson Desert, central Australia, on average abundances of a) small mammals and b) reptiles. MS = mean square, SS = sums of squares, df = degrees of freedom. Significant results (P<0.05) are shown in bold. (DOCX) [file pone.0068466.s003.docx]

**Table S3**. Repeated measures ANOVA results on the effects of historic grazing intensity (‘light’ and ‘heavy’) and recent cattle removal (‘+ cattle’ and ‘- cattle’) in the Simpson Desert, central Australia, on average abundances of a) small mammals and b) reptiles. MS = mean square, SS = sums of squares, df = degrees of freedom. Significant results (*P* < 0.05) are shown in bold.

| **a) Small mammals** | **d.f.** | **SS** | **MS** | ***F*** | ***P*** |
| --- | --- | --- | --- | --- | --- |
| Between |  |  |  |  |  |
| Grazing intensity | 1 | 10.678 | 10.678 | 10.678 | **0.037** |
| Treatment | 1 | 4.900 | 4.900 | 4.345 | 0.105 |
| Grazing intensity x Treatment | 1 | 4.444 | 4.444 | 3.941 | 0.118 |
| Error | 4 | 4.511 | 1.128 |  |  |
| Within |  |  |  |  |  |
| Trips | 4 | 93.489 | 23.372 | 25.810 | **<0.001** |
| Trip x Grazing intensity | 4 | 17.656 | 4.414 | 4.874 | **0.009** |
| Trip x Treatment | 4 | 6.489 | 1.622 | 1.791 | 0.180 |
| Trip x Grazing intensity x Treatment | 4 | 11.611 | 2.903 | 3.206 | **0.041** |
| Error | 16 | 14.489 | 0.906 |  |  |
| **b) Reptiles** | **d.f.** | **SS** | **MS** | ***F*** | ***P*** |
| Between |  |  |  |  |  |
| Grazing intensity | 1 | 15.625 | 15.625 | 3.284 | 0.144 |
| Treatment | 1 | 12.489 | 12.469 | 2.621 | 0.181 |
| Grazing intensity x Treatment | 1 | 0.803 | 0.803 | 0.169 | 0.702 |
| Error | 4 | 18.033 | 4.758 |  |  |
| Within |  |  |  |  |  |
| Trips | 2.596^a^ | 193.072 | 74.376 | 32.298 | **<0.001** |
| Trip x Grazing intensity | 2.596^a^ | 14.528 | 5.596 | 2.430 | 0.128 |
| Trip x Treatment | 2.596^a^ | 28.072 | 10.814 | 4.696 | **0.011** |
| Trip x Grazing intensity x Treatment | 2.596^a^ | 6.017 | 2.318 | 1.007 | 0.433 |
| Error | 10.384^a^ | 23.911 | 2.303 |  |  |

^a^ Greenhouse-Geisser adjusted
