# Supplementary material for: Interactions of Grazing History, Cattle Removal and Time since Rain Drive Divergent Short-Term Responses by Desert Biota
Source: PLoS One. 2013 Jul 16;8(7):e68466. doi: 10.1371/journal.pone.0068466 (PMC3713037; doi:10.1371/journal.pone.0068466)
Supplement: Table S4 — Repeated measures ANOVA results on the effects of historic grazing intensity (‘light’ and ‘heavy’) and recent cattle removal (‘+ cattle’ and ‘− cattle’) in the Simpson Desert, central Australia, on average abundances of a) Rodentia and Dasyuridae and b) Pseudomys hermannsburgensis and Sminthopsis youngsoni. Degrees of freedom for between factor tests were 1, 4 and for within factors 4, 16, for all analyses. Significant results (P<0.05) are shown in bold. (DOCX) [file pone.0068466.s004.docx]

**Table S4**. Repeated measures ANOVA results on the effects of historic grazing intensity (‘light’ and ‘heavy’) and recent cattle removal (‘+ cattle’ and ‘- cattle’) in the Simpson Desert, central Australia, on average abundances of a) Rodentia and Dasyuridae and b) *Pseudomys hermannsburgensis* and *Sminthopsis youngsoni*. Degrees of freedom for between factor tests were 1, 4 and for within factors 4, 16, for all analyses. Significant results (*P* < 0.05) are shown in bold.

| **a) Families** | **Rodentia** | | **Dasyuridae** | |
| --- | --- | --- | --- | --- |
| Source | *F* | *P* | *F* | *P* |
| Between |  |  |  |  |
| Grazing intensity | 9.468 | **0.037** | 1.742 | 0.257 |
| Treatment | 4.345 | 0.105 | 0.010 | 0.924 |
| Grazing intensity x Treatment | 3.941 | 0.118 | 0.835 | 0.413 |
| Within |  |  |  |  |
| Trips | 25.810 | **<0.001** | 6.035 | **0.004** |
| Trip x Grazing intensity | 4.874 | **0.009** | 5.860 | **0.004** |
| Trip x Treatment | 1.791 | 0.180 | 1.860 | 0.167 |
| Trip x Treatment x Grazing intensity | 3.206 | **0.041** | 3.088 | **0.046** |
| **b) Species** | ***P. hermannsburgensis*** | | ***S. youngsoni*** | |
| Source | *F* | *P* | *F* | *P* |
| Between |  |  |  |  |
| Grazing intensity | 3.738 | 0.125 | 0.857 | 0.407 |
| Treatment | 2.393 | 0.197 | 0.381 | 0.570 |
| Grazing intensity x Treatment | 1.832 | 0.247 | 2.381 | 0.198 |
| Within |  |  |  |  |
| Trips | 19.113 | **<0.001** | 3.056 | **0.048** |
| Trip x Grazing intensity | 2.605 | 0.075 | 3.955 | **0.020** |
| Trip x Treatment | 1.424 | 0.271 | 1.258 | 0.327 |
| Trip x Treatment x Grazing intensity | 2.874 | 0.057 | 2.472 | 0.086 |
