# Supplementary material for: Interactions of Grazing History, Cattle Removal and Time since Rain Drive Divergent Short-Term Responses by Desert Biota
Source: PLoS One. 2013 Jul 16;8(7):e68466. doi: 10.1371/journal.pone.0068466 (PMC3713037; doi:10.1371/journal.pone.0068466)
Supplement: Table S5 — Total captures of reptiles in sites with different historic grazing intensities (‘light’ and ‘heavy’) and recent cattle removal (‘+ cattle’ and ‘− cattle’) in the Simpson Desert, central Australia. Numbers in brackets are total captures over all trips, if different from those captured during trips when balanced datasets were obtained. Recaptures within trips have been excluded. (DOCX) [file pone.0068466.s005.docx]

**Table S5**. Total captures of reptiles in sites with different historic grazing intensities (‘light’ and ‘heavy’) and recent cattle removal (‘+ cattle’ and ‘- cattle’) in the Simpson Desert, central Australia. Numbers in brackets are total captures over all trips, if different from those captured during trips when balanced datasets were obtained. Recaptures within trips have been excluded.

|  |  |  | **Heavy grazing** | | **Light grazing** | |  |
| --- | --- | --- | --- | --- | --- | --- | --- |
|  | **Species** | | **+ cattle** | **- cattle** | **+ cattle** | **- cattle** | **TOTAL** |
| Agamids | *Ctenophorus isolepis* | | 27 | 18 | 21 | 22 | 88 (136) |
|  | *Ctenophorus nuchalis* | | 16 | 7 | 30 | 20 | 73 (135) |
|  | *Diporiphora winneckei* | | 2 | 2 | 0 | 0 | 4 (5) |
|  | *Moloch horridus* | | 0 | 1 | 0 | 0 | 1 |
|  | *Pogona vitticeps* | | 2 | 0 | 0 | 1 | 3 |
| Geckoes | *Diplodactylus conspicillatus* | | 0 | 0 | 0 | 3 | 3 (5) |
|  | *Diplodactylus stenodactylus* | | 0 | 0 | 0 | 0 | 0 (1) |
|  | *Heteronotia binoei* | | 1 | 0 | 0 | 0 | 1 |
|  | *Nephrurus levis* | | 0 | 0 | 0 | 1 | 1 (3) |
|  | *Rhynchoedura ornata* | | 1 | 0 | 0 | 6 | 7 (10) |
|  | *Strophurus ciliaris* | | 1 | 0 | 0 | 0 | 1 |
|  | *Strophurus elderi* | | 1 | 0 | 0 | 0 | 1 |
| Pygopods | *Lialis burtonis* | | 1 | 1 | 0 | 0 | 2 |
|  | *Pygopus nigriceps* | | 0 | 1 | 0 | 0 | 1 |
| Skinks | *Ctenotus ariadnae* | | 2 | 4 | 3 | 2 | 11 (20) |
|  | *Ctenotus brooksi* | | 0 | 0 | 1 | 0 | 1 |
|  | *Ctenotus calurus* | | 3 | 3 | 3 | 2 | 11 (18) |
|  | *Ctenotus dux* | | 7 | 11 | 3 | 3 | 24 (32) |
|  | *Ctenotus helenae* | | 0 | 1 | 0 | 0 | 1 |
|  | *Ctenotus leae* | | 0 | 1 | 3 | 4 | 8 (11) |
|  | *Ctenotus pantherinus* | | 13 | 7 | 17 | 6 | 43 (69) |
|  | *Ctenotus piankai* | | 1 | 3 | 0 | 1 | 5 (8) |
|  | *Egernia inornata* | | 0 | 0 | 1 | 0 | 1 |
|  | *Eremiascincus fasciolatus* | | 0 | 0 | 0 | 0 | 0 (1) |
|  | *Lerista aericeps* | | 0 | 0 | 1 | 0 | 1 (2) |
|  | *Lerista labialis* | | 26 | 18 | 61 | 38 | 143 (179) |
|  | *Menetia greyii* | | 3 | 2 | 8 | 1 | 14 (26) |
|  | *Notoscincus ornatus* | | 0 | 0 | 0 | 0 | 0 (3) |
| Varanids | *Varanus brevicauda* | | 4 | 2 | 2 | 3 | 11 (18) |
|  | *Varanus eremius* | | 1 | 1 | 0 | 1 | 3 (6) |
|  | *Varanus gilleni* | | 0 | 0 | 0 | 0 | 0 (1) |
|  | *Varanus gouldii* | | 0 | 3 | 1 | 1 | 5 (7) |
| Typhlopids | *Ramphotyphlops endoterus* | | 0 | 1 | 3 | 1 | 5 (6) |
|  | **TOTAL** | | 112 | 87 | 156 | 116 | 473 (693) |
