# Supplementary material for: Interactions of Grazing History, Cattle Removal and Time since Rain Drive Divergent Short-Term Responses by Desert Biota
Source: PLoS One. 2013 Jul 16;8(7):e68466. doi: 10.1371/journal.pone.0068466 (PMC3713037; doi:10.1371/journal.pone.0068466)
Supplement: Table S6 — Repeated measures ANOVA results on the effects of historic grazing intensity (‘light’ and ‘heavy’) and recent cattle removal (‘+ cattle’ and ‘− cattle’) in the Simpson Desert, central Australia, on average abundances of a) Agamidae and Scincidae and b) Ctenophorus isolepis, C. nuchalis, Lerista labialis and Ctenotus pantherinus. Degrees of freedom for between factor tests were 1, 4 and for within factors 4, 16, if not stated otherwise. Significant results (P<0.05) are shown in bold. (DOCX) [file pone.0068466.s006.docx]

**Table S6**. Repeated measures ANOVA results on the effects of historic grazing intensity (‘light’ and ‘heavy’) and recent cattle removal (‘+ cattle’ and ‘- cattle’) in the Simpson Desert, central Australia, on average abundances of a) Agamidae and Scincidae and b) *Ctenophorus* *isolepis,* *C.* *nuchalis, Lerista labialis* and *Ctenotus pantherinus*. Degrees of freedom for between factor tests were 1, 4 and for within factors 4, 16, if not stated otherwise. Significant results (*P* < 0.05) are shown in bold.

| **a) Families** | **Agamidae** | | **Scincidae** | |
| --- | --- | --- | --- | --- |
| Source | *F* | *P* | *F* | *P* |
| Between |  |  |  |  |
| Grazing intensity | 0.306 | 0.609 | 6.457 | 0.064 |
| Treatment | 0.618 | 0.476 | 5.520 | 0.079 |
| Grazing intensity x Treatment | 0.103 | 0.765 | 3.497 | 0.135 |
| Within |  |  |  |  |
| Trips | 12.711 | **0.007^a^** | 21.050 | **<0.001** |
| Trip x Grazing intensity | 0.514 | 0.581^a^ | 6.354 | **0.001** |
| Trip x Treatment | 1.423 | 0.299^a^ | 6.689 | **0.002** |
| Trip x Treatment x Grazing intensity | 0.091 | 0.875^a^ | 0.950 | 0.461 |

^a^ Greenhouse-Geisser adjusted (d.f. = 2, 6)

| **b) Species** | **Agamidae** | | | | **Scincidae** | | | |
| --- | --- | --- | --- | --- | --- | --- | --- | --- |
|  | ***C. nuchalis*** | | ***C. isolepis*** | | ***L. labialis*** | | ***C. pantherinus*** | |
| Source | *F* | *P* | *F* | *P* | *F* | *P* | *F* | *P* |
| Between |  |  |  |  |  |  |  |  |
| Grazing intensity | 2.065 | 0.224 | 0.011 | 0.921 | 7.254 | 0.054 | 0.818 | 0.417 |
| Treatment | 1.023 | 0.369 | 0.177 | 0.696 | 2.305 | 0.204 | 26.27 | **0.007** |
| Grazing intensity x Treatment | 0.003 | 0.960 | 0.276 | 0.627 | 0.540 | 0.503 | 2.273 | 0.206 |
| Within |  |  |  |  |  |  |  |  |
| Trips | 7.787 | **<0.01** | 14.95 | **<0.01** | 17.29 | **<0.01** | 6.292 | **0.003** |
| Trip x Grazing intensity | 2.194 | 0.116 | 0.482 | 0.749 | 7.728 | **0.001** | 4.903 | **0.009** |
| Trip x Treatment | 0.555 | 0.699 | 2.804 | 0.061 | 5.322 | **0.006** | 4.069 | **0.018** |
| Trip x Treatment x Grazing intensity | 0.555 | 0.699 | 0.804 | 0.541 | 3.058 | **0.048** | 2.847 | 0.059 |
