# Supplementary material for: Interactions of Grazing History, Cattle Removal and Time since Rain Drive Divergent Short-Term Responses by Desert Biota
Source: PLoS One. 2013 Jul 16;8(7):e68466. doi: 10.1371/journal.pone.0068466 (PMC3713037; doi:10.1371/journal.pone.0068466)
Supplement: Table S7 — Repeated measures ANOVA results on the effects of historic grazing intensity (‘light’ and ‘heavy’) and recent cattle removal (‘+ cattle’ and ‘− cattle’) in the Simpson Desert, central Australia, comparing the average species richness and diversity per grid and trip in all four treatment combinations, for a) small mammals and b) reptiles. Degrees of freedom for between factor tests were 1, 4 and for within factors 4, 16, if not stated otherwise. Significant results (P<0.05) are shown in bold. (DOCX) [file pone.0068466.s007.docx]

**Table S7**. Repeated measures ANOVA results on the effects of historic grazing intensity (‘light’ and ‘heavy’) and recent cattle removal (‘+ cattle’ and ‘- cattle’) in the Simpson Desert, central Australia, comparing the average species richness and diversity per grid and trip in all four treatment combinations, for a) small mammals and b) reptiles. Degrees of freedom for between factor tests were 1, 4 and for within factors 4, 16, if not stated otherwise. Significant results (*P* < 0.05) are shown in bold.

|  | 1. **Mammals** | | | | 1. **Reptiles** | | | |
| --- | --- | --- | --- | --- | --- | --- | --- | --- |
|  | **Richness** | | **Diversity** | | **Richness** | | **Diversity** | |
| Source | *F* | *P* | *F* | *P* | *F* | *P* | *F* | *P* |
| Between |  |  |  |  |  |  |  |  |
| Grazing intensity | 0.692 | 0.452 | 0.214 | 0.668 | 0.032 | 0.866 | 1.576 | 0.278 |
| Treatment | 1.923 | 0.238 | 0.556 | 0.497 | 1.581 | 0.277 | 1.576 | 0.278 |
| Grazing intensity x Treatment | 0.077 | 0.795 | 0.040 | 0.851 | 0.032 | 0.866 | 0.018 | 0.901 |
| Within |  |  |  |  |  |  |  |  |
| Trips | 12.71 | **<0.001** | 6.170 | **0.024^a^** | 13.89 | **<0.001** | 17.03 | **<0.001** |
| Trip x Grazing intensity | 1.571 | 0.230 | 2.199 | 0.183^a^ | 0.641 | 0.641 | 0.428 | 0.786 |
| Trip x Treatment | 2.347 | 0.099 | 2.815 | 0.119^a^ | 1.637 | 0.214 | 2.347 | 0.098 |
| Trip x Treatment x Grazing intensity | 3.531 | **0.030** | 4.302 | 0.055^a^ | 2.076 | 0.132 | 2.360 | 0.097 |

^a^ Greenhouse-Geisser adjusted (d.f. = 2, 7)
